# With Our Editors

**Published:** 1906-03-15

**Authors:** 


### WITH OUR EDITORS.

**RETROGRESSION OR ADVANCEMENT—WHICH?** The saying: "The best use a man can make of his money is to take it from his pocket and put it into his brains," seems even more applicable in these days of advancement and competition than in the days when Benj. Franklin penned the words.

No dentist can afford to allow himself to retrograde, and retrogression is sure unless he advance.

The successful merchant is ever watchful of the changes that make old goods unsalable and new varieties a necessity to meet the wants of his customers.

The successful lawyer keeps posted on changes of the laws, court decisions, etc., in order to best serve his clients.

The physician must keep posted on the advanced methods of treatment and the newer and more effective remedies to be most successful in coping with disease.

And likewise the dentist must familiarize himself with advanced methods and means in order to give his patients the best service possible.

Dentistry in its various branches is developing so rapidly that the dentist who does not keep abreast of the times will soon find himself outclassed by his fellow practitioners. And yet many dentists are careless about their reading, especially reading of dental journals.

"I haven't the time," is an excuse often given, and what an absurd excuse it is. Haven't the time to learn how to make your services of more value to your patients and yourselves; to learn how to do better work in less time; learn how to make your operations more thorough; to develop your skill; to perform newer and superior operations; to preserve the teeth for a longer time; to say nothing of the lessening of discomfort to the patient by learning humanitarian methods and putting them into practice!

At a recent dental society meeting the writer asked a certain dentist what dental journals he was taking. The astounding reply was: "I don't subscribe for any, but occasionally I get sample copies." The reply clearly showed the position that dentist occupied in the profession. And it was afterwards verified through inquiry; a second-or third rate dentist who knew a little about "fillin' teeth, and pullin' teeth, and makin' of plates."

And there are others, and many of them, who are so wise in their own conceits that they can learn nothing, or think they cannot, from the dental journals or dental societies. But such men, like Icarus, have their day.

The most successful men, and those who enjoy the largest practices, are the readers and thinkers; the progressive men who keep pace with the advancement of the profession.

Would you be content to be a mediocre, devote your spare time to the reading of popular fiction or yellow journals instead of dental literature; you will have plenty of time on your hands and it will increase as the years roll by. But would you be classed among the best, and have a practice worthy the name, keep posted in dental affairs and apply your knowledge in daily practice. No dentist ought to grudge the expenditure of five or six dollars a year for a knowledge that is worth as many hundered to him and which may be obtained by investing in five or six of the leading dental journals. Can't read them all? Then read in each that which is most suited to your needs, but take several, for no one journal contains everything of worth.

To keep thoroughly posted in dentistry is a duty every practitioner owes himself and his patients, and he who cannot afford a few dollars for such a purpose is not worthy the name of dentist. But remember that no journal, no matter how much valuable information it may contain, will benefit you unless you read it.—L. P. Bethel, in February *Summary*.

**WHO SHALL FIX THE STANDARD?** At present the standard of dental education is hard to define. If the question, "What is the standard?" be propounded, the question might be answered in a variety of ways; according to the individual belief—according to the different opinions of the two factions in the college association—or the answer might be in line with the requirements of the National Association of Dental Examiners which they are endeavoring to enforce. That there can be different answers to such a question is unfortunate. There should be only one answer. We predict that there will never be a positive and well defined standard until a number of educated men, some of whom shall be of the dental profession, shall clearly outline what shall constitute entrance requirements; curriculum and graduating merits. In other words, say what shall be the standard in dental education. The Count system when adopted (and it must be) can surely measure up to a standard —*when a standard has once been fixed*, but until then, any system of credits will simply be without an objective point or end. In the December issue of this journal we voiced the opinion that the plan proposed by Dr. A. O. Hunt at the last meeting of the Faculties' Association, viz: To constitute a Board of Regents, appointed by the Federation Internationale, the National Dental Association, and the Faculties' Association, to settle a standard in the dental educational realm, and to fix the credits given in measuring to that standard. What is the objection to Dr. Hunt's plan? We can see none, and it is plain to be seen that the examiners and the schools will not harmonize in a thousand years. They ought to, but they will not. There are men in the examining boards who cannot, it seems, treat with or speak of dental colleges without prejudice, and upon the other hand there are college men who resent any advice or requests made by the examiners. There is a woeful lack of confidence on both sides; so does it not seem best to proceed along the lines proposed by Dr. Hunt? It would not only settle standards and prevent the bickering of late years, but it would, we think, hasten reciprocity between the various States respecting interchange of license. This in turn would in time give the American dental education a standing in foreign countries, which is now denied, for it would carry weight everywhere if we could show a united and advanced front to the world through an agreed Board of Regents, elected by the highest possible authority. In foreign countries a governmental *vise* is continually being asked for. This board could confidently expect to have audience in Europe that would compel attention as if from the National Government, and many acts of injustice which are now militating against those holding the degree of D. D. S., would be prevented. We have in this country loudly demanded that the D. D. S. degree be treated with greater respect in foreign countries, and at the same time we present the spectacle of no accepted standard for that degree. The foreigner reads the attacks upon our colleges by the committee on schools of the Dental Examiners, and views the divergent efforts of Faculties' Association for higher standards. Sees one standard fixed one year and the next year reads that it is changed to the old. Reads in our journals that a diploma or license good in one state is spurned in another. Is it any wonder that a look askance is given when the graduate of an American dental college asks that recognition in Europe be given him? Let us remove the blot. Let us constitute a tribunal as heretofore outlined and present to the world something of uniformity and solidarity.—J. D. Patterson, in January *Western Journal.*

**THE PASSING OF THE "INTERNATIONAL DENTAL JOURNAL."**

In the December 1905 issue of the *International Dental Journal* announcement is officially made of its suspension of publication; the avowed reason as editorially stated being "the fact that the dentists of the first decade of the twentieth century in America are not prepared to accept, much less stand for, the principles that this journal advocates." This certainly meets the issue fairly and courageously, and we may add, in our judgment, sufficiently. But our regret is none the less sincere or deep that a journal which has done splendid service for the dental profession, which has consistently stood for the best attainment of which dentistry is capable, which regardless of criticism or of antagonism has endeavored to fulfill its purposes, should be compelled to go out of existence because of its allegiance to what seems to us to have been an impractical and utopian ideal.

What we conceive to be the fundamental error in the motive of the *International* is the variously expressed idea that there is some essential difference in the ethics of professionalism and the ethics of trade, or that the ethics of professionalism does not involve the ethics of business to precisely the same extent and degree that the ethics of business involves the ethics of professionalism. Both are based fundamentally upon common honesty and equity, and any other conception of this basic principle does not seem to us to accord with the facts. It is therefore a source of deep regret that inability or unwillingness to accept this view, and the tendency to read into professionalism something of a higher altruism which it is supposed to contain as an exclusive characteristic, has brought to at least a temporary end the career of usefulness of our highly esteemed contemporary.

Our regret is intensified by a reiteration in the valedictory editorial of what we have always felt was an unjust and unmerited criticism of that group of our professional periodical publications which the *International* has designated as "trade journals," the sole warrant for which attitude is contained in the fact that a large part of the cost of their production is borne by dental supply houses. The editor says: "The writer has no harsh word to express against our contemporaries, the so-called trade journals. Many evidences of good fellowship have been experienced from these, and while it is felt that their influence has been to the demoralization of the dental profession through the insidious poison of commercialism, they have lived up to, and been consistent with business ideals, and in their way have contributed to the practical knowledge of dentistry and thus indirectly have made it more perfect in its mechanical and scientific progress." But are these not, as a matter of fact, "harsh words?" Is it good ethics? Is it not a harsh thing to say of any man, any journal, any force in dentistry, that its influence has been to the demoralization of the dental profession through the insidious poison of commercialism? If the editor of the *Dental Cosmos* believed for one moment that the sweeping charge above quoted were true, in the smallest degree, of his own editorial activities or of the influence which his journal exerts, no power in or out of dentistry could enforce his continuance in that relation. Such evidence as is attainable on that point indicates quite another conclusion as to the influence which the periodical literature of dentistry is exerting, even though much of it is "bound up in dollar journals."—E. C. Kirk, in February *Dental Cosmos*.

**PAINLESS EXCAVATING NOT AN OPEN QUESTION.** It is interesting to note that many writers in discussing the methods of preventing pain in dental operations, take the stand that the present methods are faulty. They ask that something be presented to the profession that will do the work itself and that immediately.

Dr. Wm. Hirschfield, of Paris, France, read a paper before the American Dental Society of Europe on the subject of "Painless Excavating an Open Question," which was published in the September *Dental Review*. The Doctor has written a lengthy paper, the purpose of which has been to show that all the methods known to the profession at present are faulty, and not to be depended upon, and he has attempted to explain why these methods are not effective. The Doctor takes the same stand that the profession in general has taken in this matter. He says, among other things, "If now we may weigh free of partiality the advantages and disadvantages of all these methods (meaning pain preventing methods) we finally must come, all of us, to the one and same conclusion; an absolutely sure agent, the one which will, under all circumstances allow of immediate painless excavating without danger to the pulp, does not exist." The question of painless excavating still remains entirely open. Again he says, "We often take pride in comparing our work to general surgery; but have we forgotten that it was merely the discovery of general anesthesia which has advanced surgery to what it is in our day?" We would take issue with the Doctor when he states that painless excavating is an open question. True, as he says, no agent has been discovered that will allow immediate painless excavating in all cases, nor is there any agent that can be used that does not carry some danger with it. The surgeon has no anesthetic that will allow immediate operating, nor has he one that is free from danger, but this does not prevent him from making use of them and doing his full duty to humanity. On the other hand, the dentist has anesthetics and methods at his command, which, while not all that may be desired, makes it possible to do painless excavating, and to do it with as little danger to the tooth life as do the physician's method to the life of his patient.

Painless excavating is no longer an open question. Skilled operators are cutting ideal cavity forms in the most sensitive teeth, or removing the pulps from the same without causing one particle of pain. By skilled operators in this connection is meant men that have given much thought and time to perfecting themselves in the use of the methods at their command. If the members of the profession will but give the proper amount of time and thought to learning how to skilfully handle the methods at their command, there need be but few failures in making all operations free from pain. It is astonishing how many men, and good men, too, make utter failures when they try pain-preventing methods, simply because they lack skill and fail to use good judgment. Failing to anesthetize the sensitive tissue, they not only condemn the methods, but the men who advocate them.

Brother, if you are failing to prevent pain, it is your fault, and not the fault of the methods at command for this work. The methods given to us are not free from objection, nor are they easy of application, but when intelligently and judiciously used, they are all that could be asked.

They are difficult to apply and are attended with some danger to the pulp and patient. No surgeon gives an anesthetic, uses a knife, curette, trocar or trephine but danger is attendant, but this does not deter from duty, and humanity receives a great blessing.

So in dentistry existing methods while not all that might be desired in ease of application and freeness from danger, yet perfect results can be obtained, and when given, call blessings upon the head of the operator.

Use these methods, devoting to them half the time you spent in learning to make a perfect gold filling or a beautiful inlay, and ere long humanity will be blessing you. The difficulty of application and the danger attendant are as but naught when compared with the results obtained. Delay no longer, go seek success and if persistent great will be the reward received.—Dr. W. G. Ebersole, in January *Dentists Magazine.*

